# Degeneracy and genetic assimilation in RNA evolution

**DOI:** 10.1186/s12859-018-2497-3

**Published:** 2018-12-27

**Authors:** Reza Rezazadegan, Christian Reidys

**Affiliations:** 1 University of Virginia Biocomplexity Institute, 995 Research Park Boulevard, Charlottesville, 22911 USA; 2 Department of Mathematics, University of Virginia, 141 Cabell Drive, Charlottesville, 22904 USA

**Keywords:** RNA, Evolution, Degeneracy, Quasineutrality, Genetic assimilation, Plasticity

## Abstract

**Background:**

The neutral theory of Motoo Kimura stipulates that evolution is mostly driven by neutral mutations. However adaptive pressure eventually leads to changes in phenotype that involve non-neutral mutations. The relation between neutrality and adaptation has been studied in the context of RNA before and here we further study transitional mutations in the context of degenerate (plastic) RNA sequences and genetic assimilation. We propose quasineutral mutations, i.e. mutations which preserve an element of the phenotype *set*, as minimal mutations and study their properties. We also propose a general probabilistic interpretation of genetic assimilation and specialize it to the Boltzmann ensemble of RNA sequences.

**Results:**

We show that degenerate sequences i.e. sequences with more than one structure at the MFE level have the highest evolvability among all sequences and are central to evolutionary innovation. Degenerate sequences also tend to cluster together in the sequence space. The selective pressure in an evolutionary simulation causes the population to move towards regions with more degenerate sequences, i.e. regions at the intersection of different neutral networks, and this causes the number of such sequences to increase well beyond the average percentage of degenerate sequences in the sequence space. We also observe that evolution by quasineutral mutations tends to conserve the number of base pairs in structures and thereby maintains structural integrity even in the presence of pressure to the contrary.

**Conclusions:**

We conclude that degenerate RNA sequences play a major role in evolutionary adaptation.

## Background

An RNA molecule is a linear polymer in the nucleotides Adenine, Cytosine, Guanine, Uracil. RNA molecules play various vital roles in the cell ranging from working as messengers mediating between genes and the protein that the genes encode (coding RNA) to functioning as enzymes (non-coding RNA) [1, 2]. Moreover many of the common viruses such as SARS, Zika, Ebola and HIV are self replicating RNA molecules that evolve rapidly [3]. The nucleotide bases of an RNA molecule can form hydrogen bonds with each other. These bonds give the molecule a 3-dimensional folded structure which is believed to determine the function of non-coding RNAs [4]. Since RNA embodies both genetic code as well as biological function, it can be thought of as incorporating both a genotype (RNA sequence) and a phenotype (RNA structure). This way, RNA serves as a microcosm for studying the properties of evolution in general and of RNA viruses in particular. For example the study of RNA virus adaptation from host to host is under heavy investigation [5–7].

Since accurate prediction of this 3-dimensional structure is unfeasible at present [4], one instead studies the RNA *secondary structure* which is given by the pairs of bases that form hydrogen bonds with each other. The most widely used method for predicting RNA secondary structure *in silico* is the thermodynamic model [8] in which a free energy *E*(*σ*,*S*) is associated to each pair consisting of a sequence *σ* and a structure *S*. For a sequence *σ* the structure with minimum free energy (MFE) is proposed as the structure to which *σ* folds. There are efficient dynamical programming algorithms and software for computing the MFE structure (as well as the suboptimal structures and their energies) from the primary sequence. These include the Vienna RNA package [9] which we used for our computations in this paper.

The *folding map* which sends a sequence to its associated secondary structure can thus be regarded as the genotype-to-phenotype map for RNA. It exhibits a high degree of redundancy meaning that for each structure (satisfying a few conditions such as lack of isolates base pairs) there are many different sequences folding into it. Two RNA sequences are said to be neutral if they fold to the same structure. In general about 30% of one point mutants of an RNA sequence of length 100 are neutral. The set of all sequences folding to a structure *S* is called the *neutral network* of *S*. Neutral networks of different structures vary greatly in their sizes with some structures (usually natural structures) dominating vast portions of sequence space [10]. In general the distribution of RNA neutral networks is nearly log-normal [11–13].

This feature of RNA folding map is in perfect accord with Motoo Kimura’s *neutral theory* of evolution [14]. According to this theory, evolution is driven by neutral mutations and it is the accumulation of such mutations which results in the occurrence of beneficial mutations. This is in contrast with the selectionist view of evolution in which most mutations are either deleterious of beneficial. Neutral networks imply robustness for RNA phenotypes under mutations while at the same time increasing the number of other phenotypes to which one can evolve. Mutational robustness means that, due to the significant number of neutral neighbors for most sequences, RNA phenotypes are resilient under mutations of their underlying phenotypes. Therefore starting from a sequence in a neutral network and randomly mutating it, its structure stays the same for a period of time, as long as the mutations are neutral.

At the same time the large sizes of neutral networks result in a high degree of intertwining between various networks. In other words there are many different phenotypes to which a given one can evolve. However, as mentioned before, different RNA shapes differ in the size and shape of their neutral networks. To quantify these phenomena one defines the *mutational robustness* of a sequence to be the fraction of its one point mutants that fold into the same structure. Sequences of high robustness lie in the “interior” of a neutral network while those of low neutrality lie closer to its interface with other networks. The *evolvability* of a sequence which is the number of structures (counted without multiplicity) to which its one point mutants fold. Mutational robustness and evolvability of sequences correlate negatively [15], however there is evidence that these two quantities averaged over the sequences in a neutral network (called the robustness and evolvability of the associated phenotype respectively) correlate positively [16].

In 1985 Manfred Eigen [17] proposed the theory that, at any given time, natural selection does not result in a unique fit (or wild type) species, but a distribution of species that are closely related by mutations and whose fitness is close to one another. He termed such a distribution a *quasispecies*. Around the same time as the publication of the quasispecies theory, Gillespie [18] proposed an algorithm for the probabilistic simulation of the interactions between molecular species in a flow reactor. Even though the physics behind molecular interactions in chemistry and mutations and natural selection in biology may be very different, Gillespie’s approach can be applied to studying both [19]. Gillespie’s method was adopted by Fontana and Schuster [19] for simulating RNA evolution. Such a simulation starts with a population of RNA sequences that are chosen at random. At each step a member of the population is chosen randomly to replicate. As in actual replication in cells there is a probability, called the *error rate* that a base is changed (mutated) during replication, resulting in a mutant copy of the original sequence. One uses a fitness function to decide which members are given more chance to replicate. Fontana and Schuster use three different fitness functions: the structure distance to a given natural structure [20], minimum free energy and a sigmoid function derived from the number of helices and the number of their arcs in the structure [19].

**Fig. 1:**
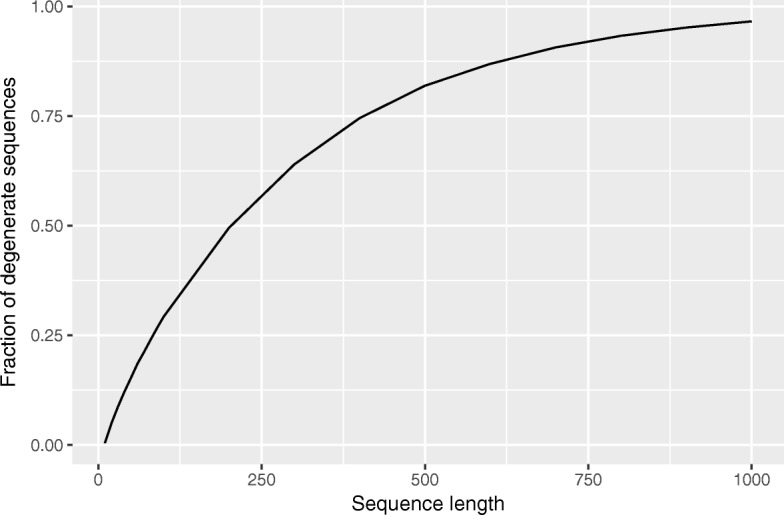
The percentage of degenerate sequences as a function of sequence length. 10^5^ random sequences of each length were used to obtain this data. The percentage of degenerate sequences is nearly a parabolic function of chain length. As sequence length increases to near 1000 almost all sequences become degenerate

One of the main observations of [20] is that if the fitness function is given by structure distance to a target structure, most of the time the population is in steady intervals in which the majority of the population folds to the same structure (called the dominant structure). Such plateaus are interspersed by times in which the dominant structure undergoes a significant change and therefore the distance to target drops, see Fig. 1. This observation gives a computational verification of the theory of *punctuated equilibria* [21].

One major caveat of the discussion thus far is having ignored the possibility that multiple phenotypes can be associated to the same genotype. *Phenotypic plasticity* is when different environmental conditions result in the association of more than one phenotype to a given genotype. It exists in biological as well as molecular species [22] and is believed to have a major role in evolutionary diversification [23]. *Structural promiscuity* is a closely related phenomenon in which a genotype, such as an enzyme, exhibits more than one function. However, unlike phenotypic plasticity, no change in the environment is needed for the extra, “promiscuous”, functions to exhibit themselves [24].

Phenotypic plasticity results in a mode of evolution called *genetic assimilation* (often confused with the Baldwin effect [25]). Genetic assimilation was defined by Conrad Waddington as a process “by which a phenotypic character, which initially is produced only in response to some environmental influence, becomes, through a process of selection, taken over by the genotype, so that it is formed even in the absence of the environmental influence which had at first been necessary” [25].

Genetic assimilation has been explained in terms of natural and artificial selection [26]. There is also a qualitative description of assimilation using dynamical (“layered”) genotype networks [27, 28]. Here, inspired by the Boltzmann ensemble of RNA (discussed below), we give a probabilistic interpretation of assimilation and then specialize it to RNA molecules. We postulate that all the phenotypes that a genotype can *possibly* have via either structural promiscuity or phenotypic plasticity belong to the abstract *phenotype space* of that genotype. For a fixed environmental condition, each element of the phenotype space has a specific probability of occurrence, with the most fit (wild type) being the most probable. However changes in the environment alter these probabilities. Such changes can either keep the wild type fixed (neutrality) or cause it to be replaced with another phenotype. However even in the former case, the probabilities of suboptimal (promiscuous) phenotypes can change. Note that the idea of a probabilistic phenotype space is somewhat similar to phenotype-genotype correlation analysis in medicine, see e.g. [29].

In this framework Waddington’s experiment can be interpreted as follows. Under normal circumstances the unusual cross-veinless phenotype exists in the phenotype space of drosophila with a small probability. The heat shock results in the probability of this phenotype to increase while still keeping the wild type fixed. However after a few generations the change in the environment results in a *neutral* evolution of the genotype to one in which both the wild type and the cross-veinless have similar probabilities. (Neutrality in the context of genetic assimilation has been discussed before, as cryptic genetic variation [30].)

We call a genotype with more than one most probable (wild type) phenotype, *degenerate*. (This terminology is somewhat different from the use of this word in biology literature.) Since in the intermediate stage of genetic assimilation a genotype (or, in our interpretation, a population of neutral genotypes) exhibits more than one wild-type phenotype, this genotype is bound to be degenerate or at least nearly degenerate in the given environment.

For RNA, the phenotype space is already given to us as the Boltzmann ensemble [31]. The Boltzmann ensemble of a sequence contains all the secondary structures compatible with it, each with a given probability given by a Boltzmann probability distribution [31]. These probabilities reflect the amount of time the molecule spends in a given folded configuration. This is in contrast with the (still prevalent) point of view that an RNA molecule has a single folded structure. The probability of a given structure is in reverse proportion with its folding (free) energy and so the structure with the minimum free energy (MFE) is the most probable one. Therefore it is often regarded as *the* structure to which the molecule folds. However, oftentimes the MFE structure is just slightly more probable than the structure(s) with the second highest probability. The dependence of the phenotype probabilities on the environment is reflected in the dependence of Boltzmann probabilities on temperature [32]. A special case of this phenomenon is RNA thermometers whose structures change as a response to changes in temperature [33].

**Fig. 2:**
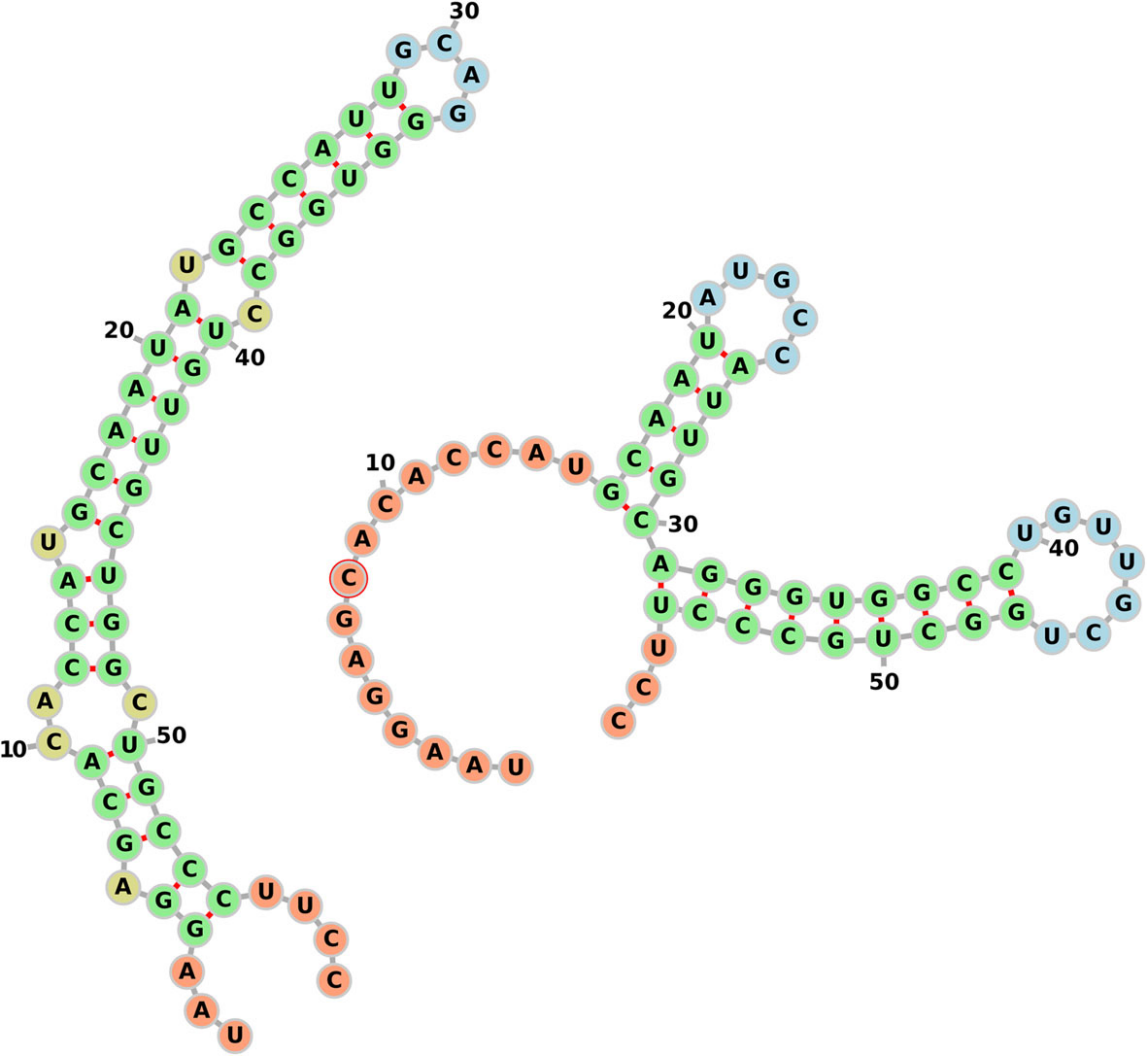
The two stable folded structures of the degenerate cIII mRNA belonging to the bacteriophage *λ* [39]. The left and the right structures have free energies are computed to be − 19.6 kc/mol and −18.1 kc/mol respectively [39]. The difference in the free energies may be due to ignoring tertiary bonds by the energy model [39]

Figure 2 gives a schematic depiction of our probabilistic interpretation of genetic assimilation in the case of RNA. In this interpretation a change first encountered in the Boltzmann ensemble later manifesting itself as a change in the genotype. This can be thought of as a special case of the duality between the sequence space and the structure space [34, 35].

**Fig. 3:**
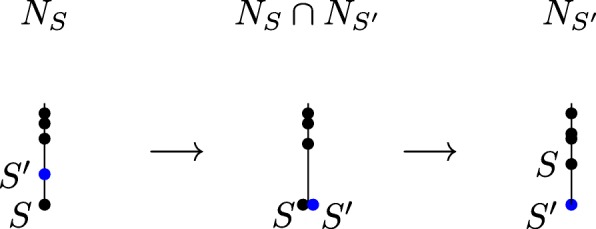
A schematic depiction of genetic assimilation through degeneracy for RNA. The vertical lines represent the energy scale in the Boltzmann ensemble of individual sequences. This means that the higher up a structure is, the higher its free energy and therefore the lower its Boltzmann probability. Thus the lowest structure is the MFE structure of the sequence. The label *N*_*S*_ denotes the neutral network of *S*. A nondegenerate RNA sequence (left) which has structure *S* at MFE level is forced by selective pressure to neutrally (cryptically) evolve into a degenerate sequence folding to two RNA structures *S*,*S*^′^ (center). Further selection causes the sequence to enter the neutral network of *S*^′^ and discard its original phenotype *S*. This way the acquired phenotypic character *S*^′^ becomes inherent to the genotype

**Fig. 4:**
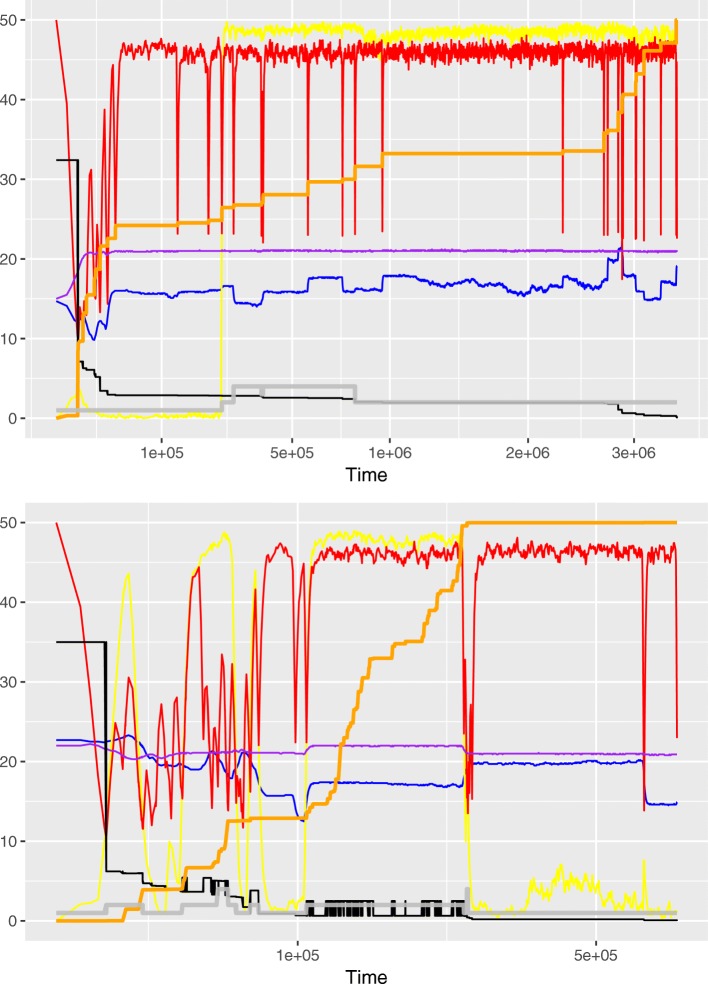
Free simulation with the single-MFE fitness function (top) and MFE-set fitness (bottom): the *x*-axis is scaled by square root. The quantities depicted are as follows. The distance of the dominant structure of the population to the target (black curve). The mean energy and mean number of base pairs in the population (the blue and purple curves respectively). The number of degenerate sequences and the number of sequences folding to the dominant structures respectively, each divided by 20 to fit in the picture (the yellow and red curves). Transitions of the dominant structure (step-wise orange curve). This curves goes up one step each time the dominant structure is changed (normalized to end at 50). The degeneracy of the parent of the current dominant structure (gray curve)

**Fig. 5:**
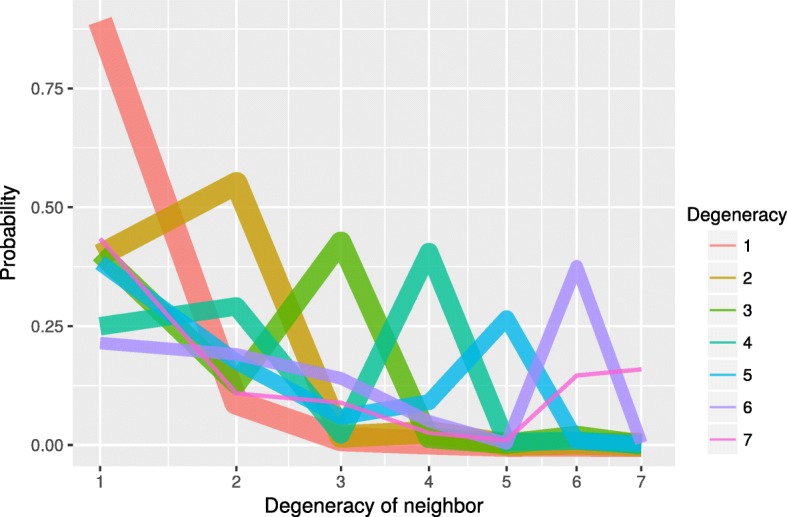
The likelihood for a sequence to have a neighbor (one point mutant) of a degeneracy *k* filtered by the degeneracy of the sequence itself. It is most likely for a sequence of degeneracy 2,3,4 or 6 to have a neighbor of the same degeneracy while for degeneracy 5, 7 (and higher odd numbers) it’s most likely to have a non-degenerate neighbor. We see that neighbors of degenerate sequences are far more likely to be degenerate compared to neighbors of nondegenerate sequences. This implies that degenerate sequences tend to form networks in sequence space

**Fig. 6:**
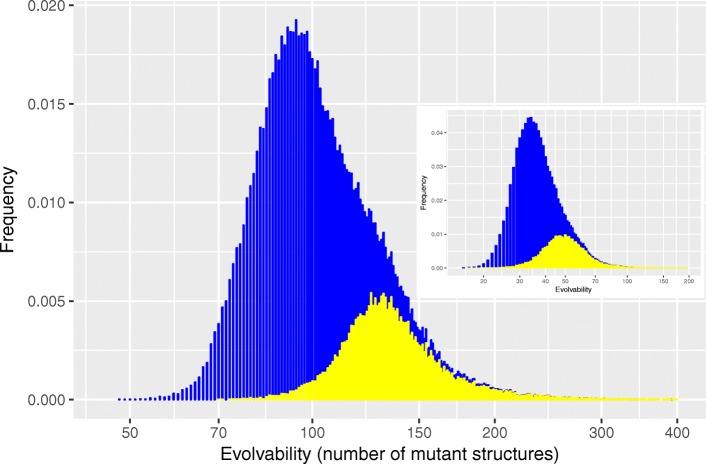
Frequency distribution of evolvability of inverse folds of Phenylalanine. 10^5^ inverse folds were obtained using a neutral walk. Among these sequences, 26491 were degenerate. The *x*-axis (scaled logarithmically) shows the total number of structures to which the one point mutants of a sequence fold. The blue bars depict the distribution for all the 10^5^ inverse folds and the yellow bars show the degenerate sequences. We observe that as degenerate sequences lie at the intersection of two (ore more) quasineutral networks, they tend to have higher evolvabilities: the mean evolvability of all the inverse folds is 111 while that of degenerate inverse folds is 143. Additionally most high-evolvability sequences are degenerate. The highest evolvability for our sample was 495. The inset shows the distribution of single-MFE evolvability, see Methods for details

A degenerate RNA sequence is one which has more than one structure at its MFE level and thus all those structures have the same (highest) probability. Examples of (nearly) degenerate RNAs include: Riboswitches, Leptomonas collosoma spliced leader RNA [36] and the untranslated leader RNA of HIV-1 virus [37]. See also Fig. 3. Degenerate sequences have high evolvability and tend to cluster together in the sequence space, see Figs. 4 and 5. As illustrated in Fig. 6, the percentage of sequences which are degenerate increases as a function of the length of the chain so that for length 1000 and longer almost all sequences are degenerate. Degenerate RNA sequences were computationally studied in [38]. In [38] we also studied *quasineutrality* which is intimately related to our interpretation of genetic assimilation in the case of RNA. Two sequences are quasineutral if they have a phenotype (MFE structure) in common and similarly a mutation relating two such sequences is called a quasineutral mutation. Such a mutation is nontrivial (non-neutral) only if one of the two sequences is degenerate. In [38] we observed that quasineutral walks i.e. walks in the sequence space in which each new sequence is a quasineutral mutant of the last, resemble neutral walks in the short run and random walks in the long run. Such walks were able to reach their targets solely by means of genetic assimilation (see also the “Results” section on quasineutral simulations). This result provides computational evidence for the genetic assimilation theory in the context of RNA evolution.

We propose the following as a possible demonstration of genetic assimilation in natural RNA. It pertains to the messenger RNA associated to the cIII gene in bacteriophage *λ*. The structure of the 5’ end of this mRNA, of length 58, is important in the regulation of translation of the associated gene [39]. In [39, 40] it is demonstrated that this mRNA posesses two different conformations (structures) referred to as A and B, see Fig. 3. When the environment temperature is increased the ratio of the two structures is shifted towards A. The authers analyse the one point mutants of this mRNA and realize that in mutants which resulted in overexpression, structure B predominates, whereas in most of the low-expression mutants, A is favored. This is because in structure B, the Shine-Dalgarno-AUG region is unpaired and, hence, more accessible for ribosome binding, whereas it is occluded in structure A. Some of these mutants have the same MFE-structure as the natural sequence and so can be regarded as being quasineutral to it.

We therefore know that increasing the temperature has almost the same effect as mutating a few of the bases (and the similarly for lowering the temp). We can re-interpret this phenomenon as fluctuations in the temperature forcing the B-sequences to turn the A-structure from a suboptimal structure to an optimal one via point mutations, thereby resulting in the degenerate mRNA.

We also investigate the effect of plasticity on evolution in the case of RNA. Whether plasticity speeds up evolution is subject to debate [41]. In [41] it is concluded that “adaptive plasticity that places populations close enough to a new phenotypic optimum for directional selection to act is the only plasticity that predictably enhances fitness and is most likely to facilitate adaptive evolution”. For RNA this has been further verified experimentally [42] and computationally [43]. (The result of [42] pertains to cryptic genetic variation however in the light of [43] it can be interpreted in terms of plasticity.) However in [15] Ancel and Fontana use simulations utilizing a fitness function obtained by averaging the fitnesses of suboptimal structures in a truncated Boltzmann ensemble to study the effect of plasticity on RNA evolution. They conclude that such a “plastic fitness function” results in the vanishing of plasticity in the population and prevents it from reaching its target. We note that from a mathematical point of view this fitness function is impossible to satisfy and the decrease in plasticity is the result of comparing a (truncated) Boltzmann ensemble with a single target structure.

To remedy this problem we use a fitness function which is given by the maximum of the fitnesses of the MFE structures of a sequence. Using the maximum gives advantage to the phenotype with higher fitness and accelerates adaptation by means of genetic assimilation which in this case is moving through the degenerate boundary of two neutral networks. As opposed to [15] our simulations reach their target. We also show that even in simulations with a simple fitness function, considering only a single MFE structure, the number of degenerate sequences rises well above the average percentage of degenerate sequences in sequence space. We show that this increase is a result of the selection pressure since degenerate sequences are highly evolvable.

We also observe that even if mutations are restricted to being quasineutral, i.e. restricting evolution only to genetic assimilation through degenerate sequences, the population still reaches its target.

## Results

### Comparison of the MFE-set and single-MFE simulations

In all our simulations the target is the Phenylalanine tRNA and fitness of a genotype is given by the secondary structure distance of its folded structure(s) to the target. MFE-set simulations take all the MFE structures of a sequence into account for measuring distance whereas single-MFE simulations use only the one picked by the ViennaRNA package, as in [20], see “Methods” section. The reason for considering two types of free simulation is to compare what we propose as more natural (MFE-set simulations) to the ones in literature [20]. In Fig. 1 we see the plots of various quantities associated with these two types of free runs. One of the first things we observe in Fig. 1 is that the MFE-set simulation reaches the target much faster than the single-MFE run.

To analyze this phenomenon further, we ran 40 simulations of either type and on average single-MFE runs took 7.3×10^6^ steps to finish whereas MFE-set runs reached the target after the average of 5.7×10^6^ steps. The standard deviations for the two quantities were 5.5×10^6^ and 3.8×10^6^ respectively with the t-test *p*-value being 0.09. This difference can be explained as follows. Since the single-MFE fitness function neglects all but one of the MFE structures of degenerate sequences, the single-MFE fitness of a sequence is always less than or equal its MFE-set fitness, see the inequality displayed in (2). In others words the latter can observe fitness in situations where the former cannot.

Only 2.6*%* of all non-neutral mutations in single-MFE simulations were quasineutral. However among the beneficial mutations (i.e. mutations that decrease the distance to the target structure, see Methods) 3.7*%* were quasineutral. Paired student t-test for the two quantities yielded a *p*-value of 0.06. This suggests that quasineutral mutations play a larger role in adaptation compared to their share of all mutations.

The spikes in the distance of the dominant structure to the target in the MFE-set run are the result of degenerate sequences dominating the population and hence two or more structures, to which these sequences fold, competing for dominance. This of course does not happen in the single-MFE runs.

In both types of simulation, the drops in the distance to the target coincide with drops in the number of sequences folding to the dominant structure. This happens when a beneficial mutation starts becoming established and the population begins to migrate to a new neutral network.

### Dominance of degenerate sequences in free simulations

In Fig. 1 we observe that the number of degenerate structures in the population is zero at the beginning however it then increases to dominate the population for a significant portion of the run time in either simulation. This happens in spite of the fact that degenerate sequences constitute only 21% of all sequences of the length 73 used in the simulations, see Fig. 6.

In order to understand this phenomenon better we ran 40 runs of each type. The highest number of degenerate sequences during each MFE-set run was at least 940. In the single-MFE runs this number was at least 700 in all but four of the runs. *Thus in almost all simulations of either type, degenerate sequences dominate the population at least at one point during the run.*

We next show that the surge in the number of degenerate sequences is a meaningful signal instead of random noise. To this end we ran simulations without a fitness function, called *non-adaptive runs*. In such a simulation all sequences have the same fitness, see Methods. In each of the non-adaptive runs the mean number of degenerate sequences was close to 210 which agrees with the percentage of the degenerate sequences in the sequence space of length 73.

The difference is seen when comparing the highest number of degenerate sequences during a run, between single-MFE and non-adaptive runs. The t-test yielded a *p*-value of less than 2.2×10^−16^ with the averages for the two types of run being 912 ± 146 and 412 ± 64 respectively. (We chose the single-MFE runs since they have a smaller number of degenerate sequences, as seen above, and therefore less likely to demonstrate a significant difference.) *This suggests that it is the selection pressure that forces the population to move towards areas of the sequence space with higher concentration of degenerate sequences.*

### The role of degenerate sequences in transitions

We next investigate the reason behind the surge in the number of degenerate sequences in simulations. We show that degenerate sequences contribute to transitions more than their share of the population.

To this end we studied the number of dominant structures with degenerate parents. Note that not all beneficial mutations result in a transition, since the result of such a mutation may die before becoming dominant. We thus recorded the degeneracy (the number of MFE structures) of the parent of each dominant structure. Note that a structure may be born and die several times before becoming dominant. Thus by the parent of such a structure we mean the last sequence that gave birth to it (i.e. introduced it to the current pool of structures) before it becoming dominant.

In Fig. 1 we see that for the majority of the length of single-MFE run and a significant portion of the MFE-set run, the parent of the dominant structure is degenerate. In general the percentage of dominant structures birthed by degenerate sequences, averaged over 40 single-MFE and MFE-set runs was 27*%*±13*%* and 56*%*±18*%* respectively. *This means that, even in single-MFE runs degenerate sequences contribute to shape innovation (transitions) more than their 21% share of the population.* The difference is much more pronounced for MFE-set simulations as they take advantage of the full MFE-set of degenerate sequences.

### The spread and evolvability of degenerate sequences

In the last subsection we demonstrated that degenerate sequences contribute to transitions more and this was possibly the reason for the surge in their numbers in the population. In this section we verify this by studying their neutrality and evolvability as well as their spread in the sequence space. In Fig. 4 we demonstrate how likely it is for a sequence of degeneracy *k* to be a neighbor of a sequence of degeneracy *l*. This plot is obtained by folding the one-point mutants of 10^5^ random sequences of length 73 (the same length as Phe-tRNA). We can see that it is most likely for a sequence to be a neighbor of a sequence of the same degeneracy, except for sequences of degeneracy 5,7,9,… which are most likely to have non-degenerate neighbors. This implies that degenerate sequences cluster together and possibly form networks in the sequence space. As a consequence once the number of degenerate sequences in the population starts to increase, it teds to accelerate.

**Fig. 7:**
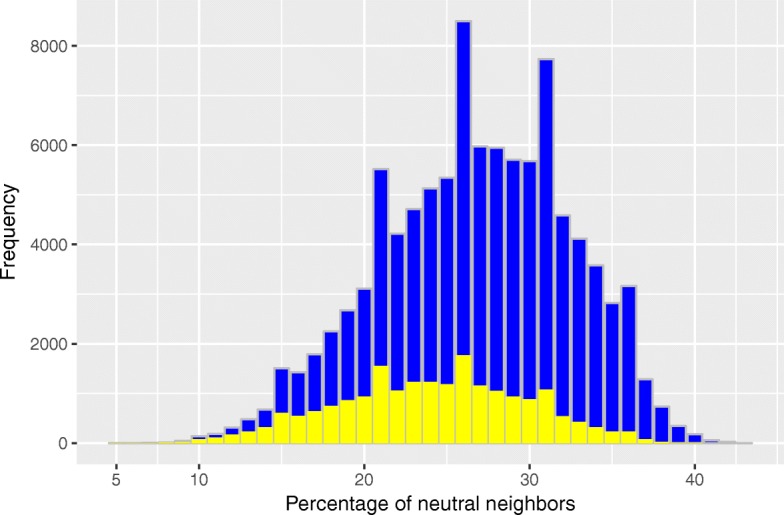
Frequency distribution of the neutrality of inverse folds of Phenylalanine. The same set of sequences is used as in Fig. 6. The *x*-axis is scaled by the square root. We compute the percentage of neutral neighbors of each such sequence with respect to Phe-tRNA i.e. the percentage of one point mutants that have Phe-tRNA structure in their MFE sets (blue bars). The distribution of neutral neighbors for the degenerate inverse folds of Phe-tRNA is depicted by the yellow bars. Most of the inverse folds of low neutrality are degenerate

What causes the number of degenerate sequences to start to increase? In Figs. 7 and 5 we plot the distributions of neutrality and evolvability in the neutral network of Phenylalanine tRNA as a representative for natural structures. We observe that degenerate sequences have the highest evolvability and lowest neutrality compared to the non-degenerate sequences. This is to be expected as degenerate sequences are on the “outposts” of the neutral network and are more “exposed” to other adjacent neutral networks. The evolvability in Fig. 5 is computed by taking all the MFE structures of each sequence into account (see “Methods” section for details). As we just demonstrated degenerate sequences have more degenerate neighbors and thus one may argue that their evolvabilities are tautologically high. However the inset in Fig. 5 depicts the distribution of evolvability computed using a single MFE structure per sequence, as done in the literature. We see that even subject to this condition, degenerate sequences exhibit maximal evolvability.

### Quasineutral simulations

**Fig. 8:**
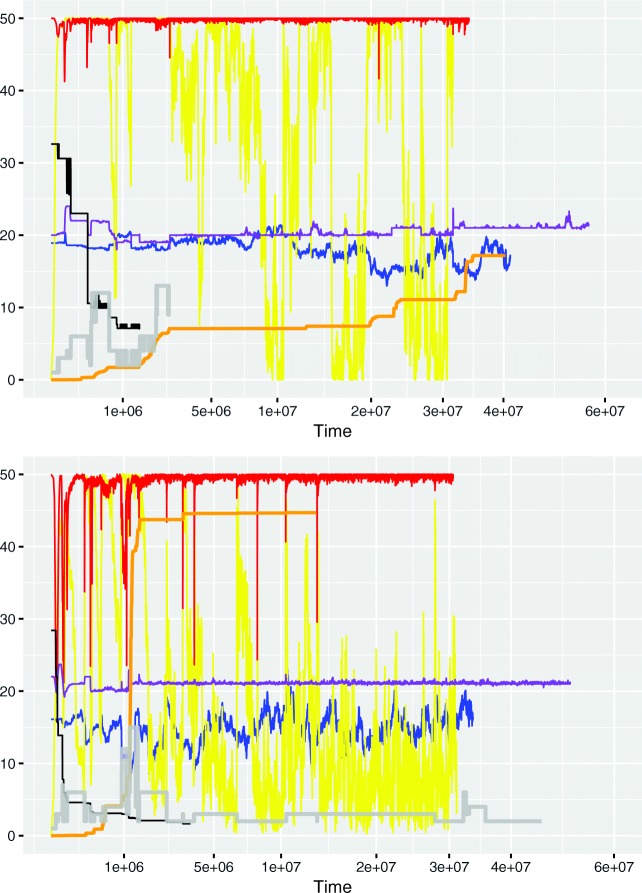
Plots of quasineutral runs with error threshold *p*=0.001 (top) and *p*=0.01 (bottom). The quantities depicted are the same as those displayed in Fig. 4

In quasineutral simulations only quasineutral mutations are allowed during replication, see “Methods” section for the technical details. In other words quasineutral simulations demonstrate how evolution would work under the minimalist constraint of having a phenotype in common in each mutation. In Fig. 8 we see plots of quasineutral runs with error rates *p*=0.001 and *p*=0.01. Because of the quasineutrality condition, in order to pass from one neutral network to another, the population has to go through the degenerate boundary between the two neutral networks. Therefore the number of degenerate sequences spikes before each transition.

Note that most quasineutral mutations are actually neutral (92% for length 100 [38]) and thus it takes much longer for a quasineutral simulation to reach its target. Moreover the ratio of the beneficial to deleterious mutations is smaller in quasineutral runs compared to the (MFE-set) free runs: 0.03 versus 0.08. We can see that in quasineutral simulations the number of sequences folding to the dominant structure is much more stable compared to the free runs.

We analyze the genotypic and phenotypic diversity of the elements of the population. There are different ways to quantify phenotypic diversity. One such quantification is to count the number of structures to which the sequences in the population fold. However degenerate sequences fold into more than one structure, therefore it is more suitable to consider the number of neutral networks in the interior of which at least one sequence lies. This is equivalent to counting the number of structures to which the non-degenerate sequences in the population fold. For the free run depicted in Fig. 1 the mean of this number is 25.7 with the maximum being 61. For the quasineutral run in Fig. 8 (top), the mean and maximum are 0.34 and 5 respectively. This shows that in a quasineutral run the sequences are more clustered and are far less spread out compared to free simulations. Moreover in the quasineutral simulation, for long periods of time all sequences are degenerate and therefore no sequence lies in the interior of any neutral network.

As a measure of the genotypic diversity of the population we consider the number of sequences folding to the dominant structure counted without multiplicity. (Note that several copies of the same sequence may exist in the population.) For the quasineutral run the mean of this number over time was 41.2 while for the free MFE-set run it was 30.2. This may be attributed to the fact that in quasineutral runs the sequences can only exit a neutral network through the minority degenerate sequences and thus they evolve neutrally inside a neutral network more than in free simulations.

### Non-adaptive runs and structure networks

A question remains at to what extent the properties of quasineutral and free runs depend on the fitness function or in other words on the selective pressure. In [20], Fontana and Schuster define a weighted directed network structure on the set of RNA secondary structures in which edge weights indicate the likelihood for the inverse folds of one structure to mutate into a sequence folding into another given structure, see Methods for details. These edge weights govern the dynamics of simulations when there is no selective pressure. Fontana and Schuster observe that the unlikely transitions between two structures for which the edge weight is minuscule, are induced by the selective pressure. Such transitions result in a significant decrease in the distance to the target.

In the “Methods” section we define the analogue of this network for quasineutral mutations which we call *quasineutral structure network*. We also introduce probabilities *ρ*(*S*,*S*^′^) and *ρ*_∗_(*S*,*S*^′^) that inform us how likely it is for an inverse fold of *S* to mutate to a sequence folding to *S*^′^ freely and quasineutrally, respectively.

The consequences of the absence of selective pressure can be studied via simulations which have no specific target and therefore all sequences have the same fitness. We refer to such simulations as “non-adaptive runs”, see “Methods” section. We ran 10 free as well as quasineutral non-adaptive runs, to observe the intrinsic differences between the two types, i.e. differences that are not caused by the selective pressure. Each such run starts with a population of 1000 random sequences.

One major difference between the non-adaptive free and quasineutral runs is the size of the pool of the dominant structure. Note that the sequence space of length 73 has more than 8.9×10^43^ elements and thus it is unexpected for a simulation starting from a random initial population to evolve into a clustered population. However it follows from general properties of stochastic processes [44] that the population indeed forms clusters. In quasineutral non-adaptive runs the size of the pool of the dominant structure is much larger than in free non-adaptive runs: an average of 890 sequences for the quasineutral run as opposed to 82 for the free run.

## Discussion

We saw that during an evolutionary simulation of a population of RNA sequences, the number of degenerate sequences rises well above their average number in sequence space, both in free and quasineutral simulations. This might be attributed the to fitness function *f*_*S*_ (see “Methods” section) that uses the minimum distance to the target of all the structures in the MFE-set of a sequence, thereby giving an implicit advantage to degenerate sequences. However as we saw in the “Results” section, the rise in the number of degenerate sequences happens even in free simulations with the fitness function *f*_*s*_ which uses only one MFE-structure per sequence, i.e. the one chosen by the Vienna RNA package. This implies that such a rise is an intrinsic property of the simulations, even of the simulations performed in [20], and indicates that degenerate sequences play a major role in adaptation. We also argued in Background that quasineutral evolution through degenerate sequences resembles genetic assimilation, see Fig. 2.

We attribute this over-representation of degenerate sequences in transitions and therefore their prominent role in adaptation to the following two facts:

- degenerate sequences have more degenerate neighbors compared to nondegenerate sequences,
- degenerate sequences are more evolvable than nondegenerate sequences.

The first fact is explained in Fig. 4 which shows us how likely it is for a sequence having *k* structures at its MFE level to have a neighbor with *l* MFE-structures. This plot indicates that degenerate sequences tend to have more degenerate neighbors. Therefore once the simulation population reaches an area of sequence space with a high density of degenerate sequences, the mutants are more likely to be degenerate themselves.

The second and more prominent fact is demonstrated in Figs. 7 and 5. We can see in those two figures that degenerate sequences have the highest evolvability and the lowest neutrality among the sequences in the neutral network of Phenylalanine tRNA. This implies that it is *plausible* for the surge in the number of degenerate sequences to be due to their higher evolvability. We however note that this surge is not observed in the “non-adaptive” simulations i.e. the ones in which all sequences have the same fitness and therefore there is no selective pressure. We thus conclude that the increase in the number of degenerate sequences is a result of both selective pressure and higher evolvability. Further experimental research is needed to fully understand the role of degenerate sequences in evolution.

Models of evolution involving genotype-phenotype maps [10] typically associate a unique phenotype to a genotype. RNA folding associating a unique minimum free energy structure to an RNA sequence is a paradigmatic example in this context. A degenerate sequence, by definition, realizes multiple phenotypes and thus has more representations and possibilities to preserve structure. This raises the question of whether the observed increase in evolvability and robustness is solely a consequence of this multiplicity.

Remarkably degenerate sequences exhibit high evolvability even if we consider only one MFE structure per sequence, see the inset in Fig. 5. Even though degenerate sequences realize multiple phenotypes and thus have more representations and possibilities to preserve structure, we can see in Fig. 7 that their neutrality (robustness) is low compared to non-degenerate sequences. Our finding concerning the observed speedup of the evolutionary optimization runs (see the “Results” section) as well as their characteristic phenotypic and genotypic diversities suggest that degenerate sequences are distinctively different from nondegenerate sequences, as they are located in the boundaries of neutral networks.

Another conclusion drawn from Fig. 5 is that ignoring the MFE-set and taking only one MFE structure into account rules out a great portion of structures that are available from a given shape by means of a mutation: the mean evolvability of sequences folding to Phenylalanine structure is 111 when using the MFE-set and is 54.4 when using a single MFE.

**Fig. 9:**
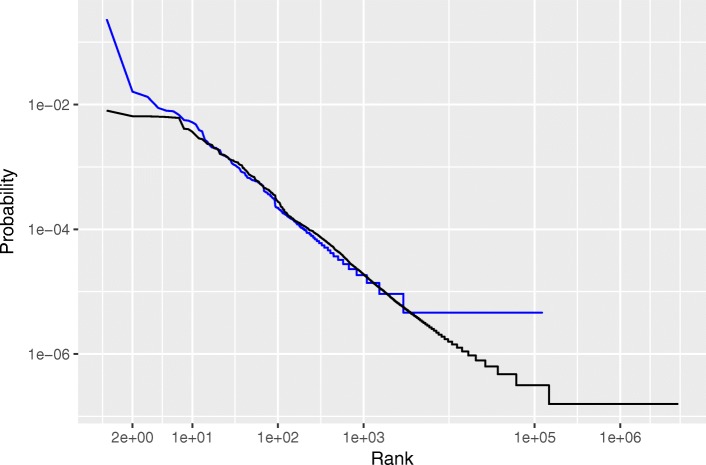
The probabilities *ρ* (black) and *ρ*_∗_ (blue) for structure neighbors of Phenylalanine. Fifty thousand inverse folds of Phenylalanine were used to compute these probabilities. Both axes are scaled logarithmically. The rank 1 structure, the structure into which Phenylalanine is most likely to mutate, either freely (black curve) or quasineutrally (blue curve) is the same for the two curves. However the probability to mutate quasineutrally to this structure, *ρ*_∗_, is 29 times higher than its free probability *ρ*. This explains why in free simulations, at the time of a transition the population splits between different neutral networks, whereas for quasineutral ones the transition is rapid and without much splitting

We saw in the “Results” section that the population in a quasineutral simulation is more genotypically diverse (i.e. has more sequences in the pool of the dominant structure, counted without multiplicities) than in a free run, while being less diverse phenotypically. We moreover observed that in the absence of the selective pressure, the pool of the dominant structure is much larger in quasineutral simulations. Some insight is provided by comparing the probability distributions *ρ* and *ρ*_∗_ (see Methods” section) for structures to which the inverse folds of Phenylalanine are likely to mutate, see Fig. 9. We can see that the initial part of the probability distribution for free mutants is much more flat compared to the distribution for quasineutral mutations. In other words in the quasineutral case there are a few select structures that dominate the distribution. This results in a much lower rate of shape innovation.

**Fig. 10:**
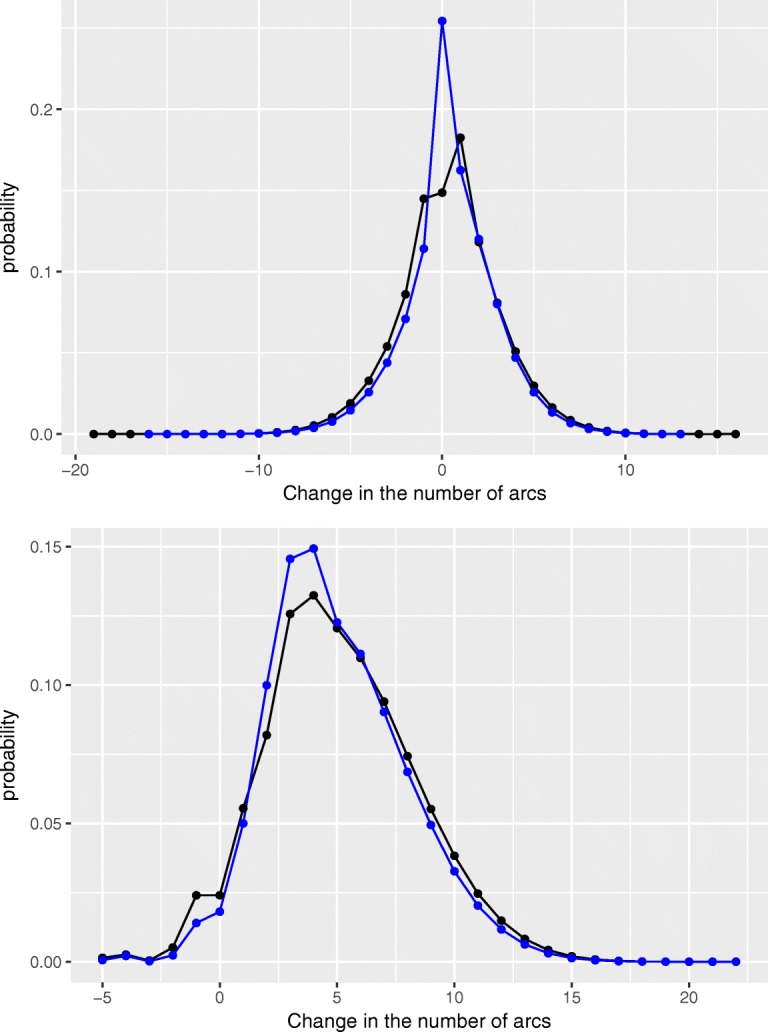
Changing the number of arcs by free (black curve) and quasineutral (blue curve) mutations. Top: Probabilities *ρ* and *ρ*_∗_ are computed for 100 randomly selected MFE structures and the average of *ρ*(*S*,*S*^′^) (respectively *ρ*_∗_(*S*,*S*^′^)) is obtained restricted to the condition that *S*,*S*^′^ differ by a given number of arcs. We observe that even though neutral mutations are excluded from the computation of both *ρ* and *ρ*^′^, quasineutral mutations have a much higher likelihood of preserving the number of arcs. The probability of a positive (resp. negative) change in the number of arcs for free mutations is 0.5 (resp. 0.35) and for quasineutral mutations it is 0.46 (resp. 0.28). This means that quasineutral mutations are slightly less likely to decrease the number of arcs. Bottom: The same probabilities for structures *S* with 4 or 5 arcs. We see that when the number of arcs is low, quasineutral simulations are less likely to decrease the number of arcs and so to result in the structure to mutate to an open chain

It is important to note that the choice of secondary structure metric plays a role in the behavior of simulations. To demonstrate this, we ran simulations in which the base pair distance was used to measure the distance of structures to the target. The base pair distance between two structures *S* and *S*^′^ is given by the number of base pairs present in *S*_1_ but not in *S*_2_ plus the number of ones present in *S*_2_ but not in *S*_1_. So one can expect that in a simulation using this metric, the series of dominant structures connecting the initial structure *S*_0_ to the target *S*_*T*_ is given by removing the arcs that are in *S*_0_ and not in *S*_*T*_ and adding the arcs that are in *S*_*T*_ and not in *S*_0_. We observe that this is indeed the case and in 18.5*%* of the 10^3^ free simulations using base pair distance, the average MFE of the population drops to zero, i.e. the open chain becomes dominant. However the quasineutral runs even with base pair distance avoided this phenomenon. This can be attributed to the fact that quasineutral mutations tend to maintain the number of base pairs, see Fig. 10. Quasineutral mutations thus preserve the integrity of the structures involved even when there is a strong pressure to reduce the number of base pairs.

## Methods

### Secondary structure metrics

Secondary structure metrics are used for measuring the dissimilarity of structures of the same length. Let *s* be the dot-bracket notation of a secondary structure. To *s* we associate a vector *v*_*s*_ as follows. First define *f*(*i*) to be zero if *s*[*i*] is a dot, $\frac {1}{l-i}$ if *s*[ *i*] is the start of an arc that ends at *l* and $\frac {1}{i-l}$ if *s*[ *i*] is the end of an arc that started at location *l*. One associates a vector to *s* whose *k*’th entry is given by $v[k]=\sum _{i\leq k} f(k)$. The weighted Motzkin (or Mountain) distance [45] between two structures is then given by

1 $$ d_{WM}(s,s')=\sum_{i} |f(i)|.  $$

In contrast to the Motzkin distance, the *d*_*WM*_-distance between the open chain and any structure with only one arc is the same. Tree edit distance is widely used for comparing secondary structures such as in the simulations done by Fontana and Schuster. However it is slow to compute as one needs to find the shortest edit path connecting the trees associated to the two structures. According to [45] *d*_*WM*_ and the tree-edit distance exhibit similar distributions of (normalized) distances between random structures. Therefore *d*_*WM*_ represents a suitable alternative to the tree-edit distance when comparing a large number of secondary structures.

### Degenerate sequences and quasineutrality

Following [38], an RNA sequence *σ* is *degenerate* if it has more than one structure at the minimum free energy level. The set of MFE-structures is called the MFE-set of the sequence and denoted by MFE(*σ*). The cardinality of this set is the *degeneracy* of the sequence. In the ViennaRNA package [9], one element of the MFE-set is chosen at random and called the MFE-structure of the sequence. We denote the latter by MFE_*σ*_. Two sequences are called *quasineutral* if their MFE-sets have non-empty intersection, or in other words if they share a phenotype. They are called *neutral* if their MFE-sets are identical. A quasineutral mutation of *σ* is a sequence *σ*^′^ of Hamming distance one to *σ* which is quasineutral to *σ*. For example two sequences with MFE-sets {*S*} and {*S*,*S*^′^} are quasineutral but not neutral.

We call the set of all sequences which have a structure *S* in their MFE-sets the *neutral network* of *S* and denote it by *N*_*S*_. We refer to the set of non-degenerate and degenerate sequences in *N*_*S*_ as the *interior* and the *boundary* of the neutral network^1^. By construction the sequences in the boundary of *N*_*S*_ belong to (the boundaries of) other neutral networks as well.

### Neutrality and evolvability

The *neutrality* (or mutational robustness) of an RNA sequence *σ* is the percentage of its one point mutants that fold into the same structure as *σ*. Since in this paper we take the multiplicity of MFE-structures into account, as in [38] we consider neutrality *with respect to a given structure S*. This means that both *σ* and its mutant have *S* in their respective MFE-sets. The *evolvability* of *σ* is on the other hand is the number of all structures two which the one point mutants of *σ* fold. These structures are counted without multiplicity i.e. a structure appearing in the MFE-sets of several one point mutants is counted only once.

For the purpose of comparison we also consider the *single-MFE evolvability* i.e. instead of all the elements of the MFE-set, only the one chosen by the ViennaRNA package as the MFE-structure is counted for each sequence.

### Free and quasineutral simulations

We conducted two different types of population simulations: free simulations similar to those of [20] in which there is no restriction on mutations and the quasineutral simulations in which mutations are restricted to quasineutral ones. For all our simulations the Phenylalanine tRNA (of length 73) is the target. The initial pool of sequences is populated by 1000 copies of a randomly chosen sequence.

In an experimental setting the fitness of an RNA genotype may be assessed by its binding capacity to a target site or, in the case of RNA viruses, its adaptability to a new host. Here however, as in [20], our goal is to study evolutionary trajectories instead of the end result. Therefore we consider fitness to be given by the secondary structure distance to a given target structure. Thus whether a mutation is deleterious or beneficial depends on whether it reduces the distance to the target or increases it. As in [20] we only consider point mutations and ignore additions and deletions. This way, by restricting the simulations to sequences of the same length, we keep the techniques simple while retaining enough of the complexity of the problem at hand.

For the free runs two different types of fitness function were considered: the single-MFE fitness and the MFE-set fitness functions. In both cases the fitness of a member *σ* of the population is defined to be the reciprocal of the secondary structure distance *d* of its MFE-structure to the target structure. One has a choice of whether to take all the structures in the MFE set MFE(*σ*) of a sequence *σ* into account for measuring this distance or not. In the former case fitness of a sequence is defined to be the maximum fitness of its MFE-structures:

$$f_{S}(\sigma)=\left[{\min_{S\in \text{MFE}(\sigma)}d(S, \text{Phe})}\right]^{-1}. $$

We refer to this fitness function as the *MFE-set fitness function*. The reason for using the maximum is that a degenerate sequence lies at the intersection of the neutral networks of all the structures in its MFE-set and so is able to mutate to a sequence in each one of them.^2^ In the latter case, as in [20] one only employs the ViennaRNA MFE-structure MFE_*V*_(*σ*) to measure fitness:

$$f_{s}(\sigma)=\bigl[{d(\text{MFE}_{V}(\sigma), \text{Phe})}\bigr]^{-1}. $$

We call this function the *single-MFE fitness function*. This method is equivalent to choosing an MFE structure from the MFE set at random and measuring its distance to the target. By construction for each sequence *σ* holds

2 $$ f_{S}(\sigma)\geq f_{s}(\sigma).  $$

A sufficient condition for equality is non-degeneracy of *σ* i.e. having only one structure at the MFE level. This condition is however not necessary. As we will see *f*_*S*_ results in more efficient simulations.

At each step a member *σ* of the population is chosen at random to replicate. The members are always ranked by their fitness and the likelihood to be chosen is linearly proportional to rank. As in [20] we chose the replication error rate to be *p*=10^−3^, meaning that during replication, each base is mutated with the probability of 10^−3^. A mutation from a sequence *σ* to *σ*^′^ is called *beneficial* if *σ*^′^ is more fit than *σ*.

In free runs no condition is imposed on this (possibly mutated) copy. However in the quasineutral simulations we only want the quasineutral mutations to contribute to evolution therefore, after a sequence is chosen for replication, it is replicated until a quasineutral mutant is obtained. Only this quasineutral mutant is added to the pool. For quasineutral runs we use the MFE-set fitness function *F*_*S*_ only.

By the number of steps we mean the number of replications in the simulation. In the quasineutral runs the (rejected) non-quasineutral mutations do not count towards the number of steps. After each replication a random member of the population (regardless of its fitness) is removed to keep the population constant. At any given step the structure pool of the population is the set of all structures (counted without multiplicity) to which the population folds. In the *f*_*M*_-simulations all the MFE-structures of each sequence contribute to the pool whereas in the *f*_*V*_ runs only the Vienna MFE-structure contributes. The *dominant structure* is the one into which the majority of the population folds. Note that in *f*_*M*_-runs, degenerate sequences can cause the majority of the population to fold to more than one structure at once. A *transition* is a step at which the dominant structure changes to a structure which has not been dominant before.

Simulations terminate as soon as the target structure becomes the dominant structure.

### Non-adaptive simulations

To see whether the increase in the number of degenerate sequences is caused by adaptive pressure or not, we ran non-adaptive simulations. The free or quasineutral non-adaptive simulations are the same as the free and quasineutral simulations discussed above except that in the former all members of the population have the same fitness. This means that a population of RNA sequences mutates freely (in free simulations) or quasineutrally (in quasineutral simulations) and choosing for replication is completely random. The population is kept constant as in targeted simulations.

### Free and quasquasineutral networks of structures

In [20] Fontana and Schuster consider a directed edge-weighted network whose vertices are given by RNA structures of a given length. The edges and their weights are constructed as follows: for a structure *S* one considers a sample *Σ* of inverse folds of *S* and list all the structures to which the one point mutants of the elements of *Σ* fold. If *S*^′^ is a structure obtained in this way then they consider the weight $\bar \rho (S,S')$ which is the fraction of the inverse folds *σ*∈*Σ* that have at least one one-point mutant that folds into *S*^′^.

For each *S* they then sort these structures *S*^′^ according to their weights

3 $$ S'_{1}, S'_{2}, S'_{3},\ldots.  $$

$\bar \rho (S,S'_{n})$ as a function of *n* drops very fast and they choose a threshold *ε* and consider a directed edge from *S* to each $S^{\prime }_{i}$ for which $\bar \rho (S,S'_{i})>\epsilon $. We refer to this directed graph as the *free structure network*.

They use these likelihoods to decide whether a transition in a simulation is continuous or discontinuous: a transition from *S* to *S*^′^ is continuous if the weight of the edge from *S* to *S*^′^ is large compared to the other edges emanating from *S*. In practice in [20] continuity of a transition is decided by whether the new sequence already existed in the structure pool before the transition or not. This is because if *S*_2_ existed in the pool when *S*_1_ was dominant then it is likely that the mutants of the sequences folding to *S*_1_ fold to S_2_ and vice versa.

In [20] the authors compute the outward star (the *characteristic set*) of the Phenylalanine tRNA in this graph. Moreover it is observed in [20] that the majority of edges are given by lengthening or shortening a stack or destroying a whole stack element. The edge weights in this graph, between structures *S* and *S*^′^, give us the likelihood that a free simulation would move from being dominated by *S* to being dominated by *S*^′^.

In this paper we consider a variant of this graph in which the edge weights, denoted by $\bar \rho _{*}(S,S')$, are given by the fraction of inverse folds of *S* which have at least one *quasineutral* one-point mutant which folds into *S*^′^. One then uses the same method as above to discard structures *S*^′^ of low weight and build a directed graph which we call *quasineutral structure network*.

Note that since only a fraction of one-point mutants of a sequence are quasineutral (on average 8% for length 100 [38]), for a given pair of structures *S*,*S*^′^, $\bar \rho _{*}(S,S')$ is usually much smaller than $\bar \rho (S,S')$. In order to compare the two quantities we take the quotient

$$ \rho(S,S')=\frac{\bar\rho(S,S')}{\sum_{S^{\prime\prime}\neq S} \bar\rho(S,S^{\prime\prime}) }. $$

We set *ρ*(*S*,*S*)=0. In contrast to $\bar \rho $, the sum $\sum _{S'} \rho (S,S') $ equals one and so *ρ*(*S*,*S*^′^) computes the probability of entering the neutral network of *S*^′^, by means of one-point mutations, when leaving the neutral network of *S*.

We obtain *ρ*_∗_(*S*,*S*^′^) from $\bar \rho _{*}(S,S')$ in a similar way. Since $\sum _{S'} \rho (S,S')= \sum _{S'} \rho _{*}(S,S') =1 $, we can compare *ρ*(*S*,*S*^′^) and *ρ*_∗_(*S*,*S*^′^) to see how the likelihood of passing from *S* to *S*^′^ by means of a single free vs quasineutral mutation are different. Note that since most quasineutral mutations are neutral (about 92% for sequences of length 100), in general $\bar \rho _{*}(S,S)$ is much larger than $\bar \rho (S,S)$.

## Conclusion

We showed that degenerate sequences have the highest evolvability among all sequences and are central to evolutionary innovation. This is important given that the multiplicity of folded structures of RNA sequences is generally ignored.

The selective pressure in an evolutionary simulation causes the population to move towards regions with more degenerate sequences and this causes the number of such sequences to increase well beyond the average percentage of degenerate sequences in the sequence space. We conclude that degenerate RNA sequences play a major role in evolutionary adaptation and further experimental research is needed to better understand this role.

## Abbreviations

MFE: Minimum free eneegy
RNA: Ribonucleic acid
tRNA: Transfer RNA

## Footnotes

1 This terminology is slightly different from that of [38].

2 Using the mean function would force the population to enter the neutral network of the target structure through non-degenerate sequences.
